# Comparative proteomic analysis of the angiogenic response in an in vitro model of vascular endothelial cells to excretory/secretory and somatic antigens of *Dirofilaria immitis*

**DOI:** 10.1186/s13071-026-07307-2

**Published:** 2026-08-20

**Authors:** Manuel Collado-Cuadrado, Alfonso Balmori-de la Puente, Ana Montero-Calle, Sara Vázquez-Ávila, Rodrigo Barderas, Javier Sotillo, Elena Carretón, Miguel Pericacho, Rodrigo Morchón

**Affiliations:** 1 Zoonotic Diseases and One Health Group, Faculty of Pharmacy, Centre for Environmental Studies and Rural Dynamization (CEADIR), University of Salamanca, 37007 Salamanca, Spain; 2 Chronic Disease Programme, UFIEC, Instituto de Salud Carlos III, 28220 Majadahonda, Madrid, Spain; 3 Proteomics Core UCCTs, Instituto de Salud Carlos III, Madrid, Spain; 4 Reference and Research Laboratory in Parasitology, Centro Nacional de Microbiología, Instituto de Salud Carlos III, Madrid, Spain; 5 CIBER of Frailty and Healthy Aging (CIBERFES), 28029 Madrid, Spain; 6 Internal Medicine, Faculty of Veterinary Medicine, Research Institute of Biomedical and Health Sciences (IUIBS), University of Las Palmas De Gran Canaria, Las Palmas, Spain; 7 Department of Physiology and Pharmacology, University of Salamanca, 37007 Salamanca, Spain; 8 Biomedical Research Institute of Salamanca (IBSAL), University of Salamanca, Salamanca, Spain

**Keywords:** Excretory/secretory antigens, Somatic antigens, *Dirofilaria immitis*, Endotelial cells, Proteomic, Angiogenic process, Heartworm

## Abstract

**Background:**

*Dirofilaria immitis* infection induces endothelial inflammation and vascular remodeling, contributing to heartworm disease pathology. While excretory/secretory antigens (DiES) are known to promote angiogenesis, somatic antigens (DiSA) exhibit different modulatory effects. This study aims to characterize the differential angiogenic response of endothelial cells to these antigenic fractions using a proteomic approach.

**Methods:**

Human umbilical vein endothelial cells were cultured and stimulated for 24 h with DiES or DiSA, either alone or supplemented with vascular endothelial growth factor (VEGF)-A to simulate active angiogenesis. Proteomic profiles of cell supernatants and lysates were analyzed using data-independent acquisition-label-free quantification (DIA-LFQ) liquid chromatography coupled to tandem mass spectrometry (LC–MS/MS). Bioinformatic analyses, including Gene Ontology and pathway enrichment, were performed to identify dysregulated proteins and molecular mechanisms related to angiogenesis.

**Results:**

Stimulation with DiES, particularly in combination with VEGF-A, produced the most significant enrichment of angiogenic processes, characterized by the downregulated anti-angiogenic proteins tryptophanyl-tRNA synthetase 1 (WARS1) and the modulation of the phosphatidylinositol-3 kinase (PI3K)/protein kinase B (AKT) pathway [upregulated 3-phosphoinositide-dependent protein kinase 1 (PDPK1) and downregulated AKT3]. Conversely, DiSA induced a limited angiogenic response with fewer dysregulated proteins. Notably, angiopoietin-2 (ANGPT2) was upregulated in all treatments, suggesting universal vascular destabilization. However, in the absence of VEGF-A, both antigens induced markers of vascular regression and cell lysis.

**Conclusions:**

DiSA and DiES exert distinct immunomodulatory effects on the endothelium. DiES acts synergistically with VEGF-A to drive angiogenesis via the ANGPT/tyrosine kinase receptor (TIE) and PI3K/AKT axes, whereas DiSA fails to support this process effectively. These proteomic insights contribute to elucidate the molecular mechanisms driving vascular remodeling and pathology in heartworm disease.

**Graphical Abstract:**

**Figure:**
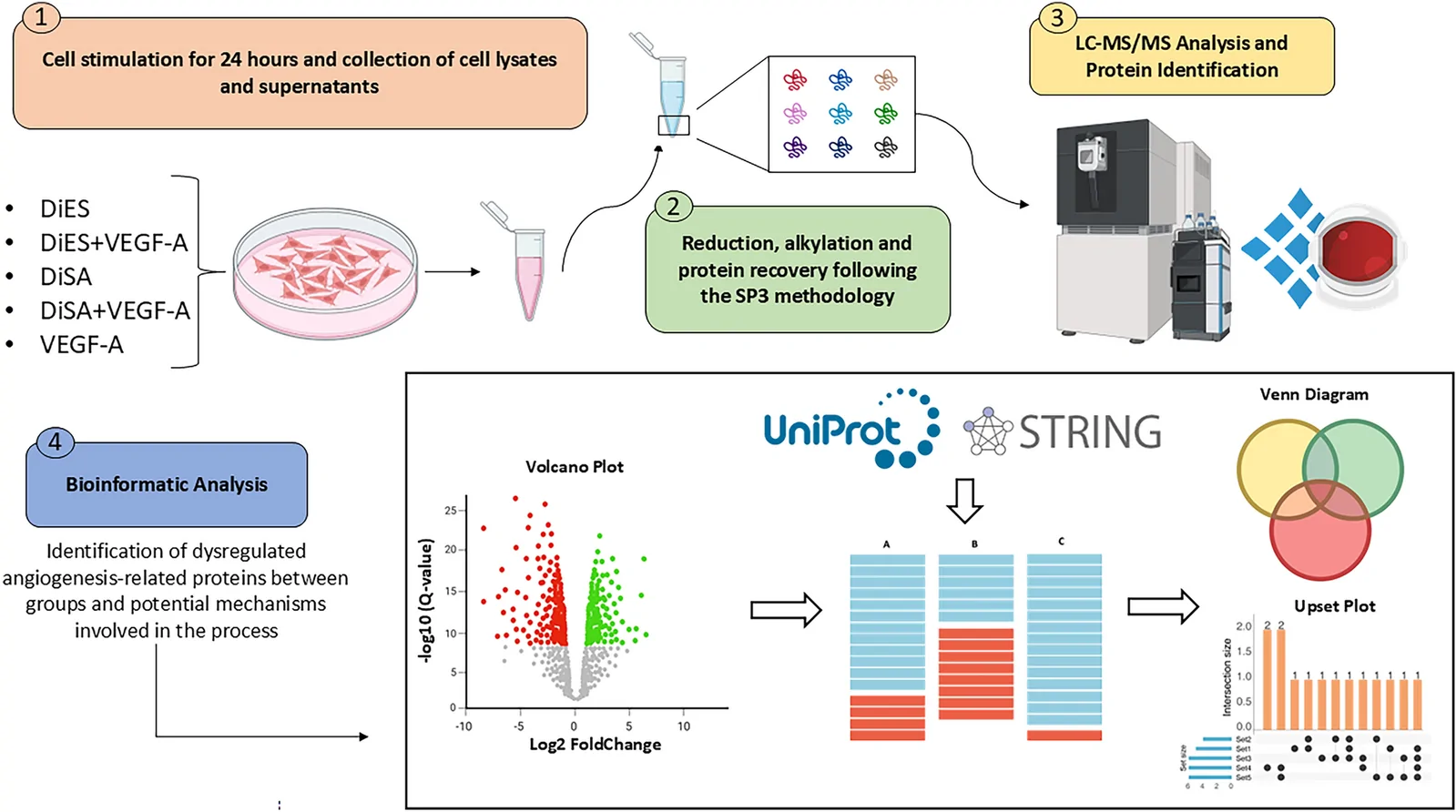

**Supplementary Information:**

The online version contains supplementary material available at 10.1186/s13071-026-07307-2.

## Background

Angiogenesis, the process by which new blood vessels are formed from preexisting ones, is essential for development, tissue repair, and wound healing, as well as for chronic inflammation, cancer, and parasitic infections [1, 2]. Its regulation mainly depends on vascular endothelial growth factors (VEGF), particularly VEGF-A, which promotes endothelial cell proliferation, migration, and survival through activation of the VEGFR-2 receptor [3]. Other mediators, such as fibroblast growth factors (FGF), angiopoietins, and matrix metalloproteinases, modulate the balance between pro- and anti-angiogenic signals [4].

In parasitic infections, the vascular endothelium acts as an immunomodulatory organ that can either promote neovascularization or counteract vascular injury [5]. Several helminths and protozoa have been shown to alter host angiogenesis through mechanisms including hypoxia, oxidative stress, and the secretion of parasite-derived proteins that mimic host molecules [6, 7].

*Dirofilaria immitis* (Leidy, 1856), the etiological agent of cardiopulmonary dirofilariosis (heartworm disease), is a filarial nematode that resides primarily in the pulmonary arteries and right ventricle of dogs and cats. In humans, the parasite does not reach the adult stage and is therefore considered an accidental host. This disease primarily affects the vascular and pulmonary systems, where adult worms induce endothelial irritation, inflammation, and vascular remodeling, contributing to pulmonary hypertension and cardiac damage [8]. In addition to the mechanical damage caused by the worms, parasite-derived antigenic products modulate host coagulation, fibrinolysis, and endothelial responses [8, 9].

Excretory/secretory antigens of *D. immitis* (DiES) are able to bind host plasminogen and promote its activation, stimulating extracellular matrix degradation and cell migration [10]. These antigens also induce the expression of pro-angiogenic factors such as VEGF-A and endoglin, highlighting their role in promoting vascular growth [11]. In contrast, somatic antigens (DiSA) show a different pattern of endothelial activation. Although VEGF-A production increases, capillary-like structure formation is not induced, suggesting a modulatory or even anti-angiogenic effect [12]. Furthermore, antigens derived from the endosymbiotic bacterium *Wolbachia* have been reported to contribute to the inhibition of angiogenesis [13, 14]. At the molecular level, several DiES proteins, including glyceraldehyde-3-phosphate dehydrogenase (GAPDH), fructose-bisphosphate aldolase (FBAL), actin (ACT), and galectin (GAL) have been associated with pro-angiogenic and profibrinolytic activities, stimulating endothelial and vascular smooth muscle cell proliferation and migration, as well as pseudo-capillary formation [15, 16].

The aim of this study was to perform a comparative proteomic analysis using an in vitro model of vascular endothelial cells to identify proteins and molecular pathways differentially regulated in relation to the angiogenic process, distinguishing between the pro- and anti-angiogenic responses induced by two antigenic fractions of the parasite (somatic and excretory/secretory antigens). The results obtained will contribute to a better understanding of endothelial modulation induced by *D. immitis* and its role in the vascular pathophysiology of heartworm disease, providing potentially valuable insights for the development of novel diagnostic and therapeutic strategies.

## Methods

### Antigen preparation

DiSA and DiES were obtained following the methodology previously described by Morchón et al. [17] and González-Miguel et al. [18], respectively, with some modifications. In brief, DiSA was prepared with 6 live worms (2 male and 4 female) and DiES was prepared with 23 live worms (8 male and 15 female). In all cases adult worms were obtained from naturally infected dogs from the Veterinary Teaching Hospital of the University of Las Palmas de Gran Canaria. Protein concentration was measured using the detergent-compatible (DC) protein assay commercial kit (Bio-Rad Laboratories, Hercules, CA, USA). The resulting samples were stored at −80 °C until analysis.

### Cell culture and stimulation of endothelial cells

An in vitro model of human umbilical vein endothelial cells (HUVEC) was used. Cells were maintained in a CO_2_ incubator (Thermo Fisher Scientific, Spain) at 37 °C, 95% humidity, and 5% CO_2_, in plates previously coated with a matrix solution containing 0.1% porcine gelatin (Sigma Chemical Co., St. Louis, MO, USA), 0.01% fibronectin (Sigma-Aldrich, St. Louis, MO, USA), and 0.01% collagen (Corning, NY, USA) [11, 14–16]. Cells were cultured in Endothelial Basal Medium-2 (Lonza, Walkersville, MD, USA) supplemented with SingleQuots® (Lonza, Walkersville, MD, USA): 10 mL FBS, 0.5 mL heparin (22.5 μg/mL), 0.5 mL VEGF (0.5 ng/mL), 0.5 mL ascorbic acid (1 μg/mL), 2 mL hFGF-B (10 ng/mL), 0.2 mL hydrocortisone (0.2 μg/mL), 0.5 mL hEGF (5 ng/mL), 0.5 mL GA-1000 (Gentamicin 30 mg/mL; Amphotericin B 15 μg/mL), and 0.5 mL R3-IGF-3 (20 ng/mL). The culture medium was changed every 3 days.

Cell expansion was performed by trypsinization (Trypsin/EDTA, Lonza, USA) when endothelial cell monolayers reached nearly 100% confluence. Treatments were carried out in 60-mm plates containing confluent endothelial cells exposed to 1 μg/μL of VEGF-A, DiSA, DiES, DiSA + VEGF-A and DiES + VEGF-A for 24 h [11, 12]. Non-stimulated cells were used as controls under the same conditions. The culture medium supernatants and lysates from vascular endothelial cell cultures were collected at 4 °C and stored at −80 °C [11, 12]. All experimental conditions were performed in triplicate.

### Cellular viability and cytotoxity assays

Subsequently, cell counts were performed using the equipment Countess^®^ Automated Cell Counter (Invitrogen) following the manufacturer’s instructions to analyze the cellular viability (living and dead cells after treatment). Cytotoxicity was assessed in the supernatant of the stimulated and control cell cultures by the Toxilight BioAssay Kit (Cambrex, Verviers, Belgium) following commercial instructions. This commercial kit quantitatively measures the release of adenylate kinase from damaged cells.

### Protein sample processing

Each protein sample (20 µg in 100 µL of RIPA buffer) was subjected to sequential reduction, alkylation, and enzymatic digestion. Proteins were reduced by adding 11 µL of 100 mM tris(2-carboxyethyl)phosphine (TCEP) (PanReac AppliChem, Barcelona, Spain) and incubating at 37 °C for 45 min under gentle agitation (600 rpm). Subsequently, 12 µL of 400 mM chloroacetamide (PanReac AppliChem) were added for alkylation and the samples were mixed at 600 rpm for 30 min in darkness to promote denaturation and enhance subsequent proteolysis.

Protein immobilization was achieved by adding 100 µL of a mixture of Sera-Mag magnetic beads (hydrophilic and hydrophobic) and 200 µL of 100% acetonitrile (PanReac AppliChem). Samples were incubated for 35 min at room temperature with continuous shaking (600 rpm) to allow for protein adsorption onto the beads. The tubes were then placed on a magnetic rack for 2 min, the supernatant was discarded, and the pellet was washed twice with 200 µL of 70% ethanol and once with 200 µL of pure acetonitrile for 30 s each. The beads were air-dried for approximately 30 s and resuspended in 100 µL of digestion buffer containing 20 µg of sequencing-grade trypsin in 2 mL of 200 mM ammonium bicarbonate (pH 8.0; PanReac AppliChem). Digestion proceeded for 14 h at 37 °C with orbital shaking (600 rpm).

After incubation, samples were sonicated and the supernatant was collected. Beads were then resuspended and sonicated again in 100 µL of ammonium bicarbonate to maximize peptide recovery. Both supernatants were pooled, yielding a final volume of 200 µL, vacuum-dried, and stored at −20 °C until LC–MS/MS analysis.

### Data-independent acquisition-label-free quantification (DIA-LFQ) LC–MS/MS analysis

Peptide mixtures were analyzed by liquid chromatography coupled to tandem mass spectrometry (LC–MS/MS) on an Orbitrap Astral mass spectrometer interfaced with a Vanquish Neo UHPLC system (Thermo Fisher Scientific). Peptides were first trapped on a PepMap Trap Cartridge (5 µm, 300 µm × 5 mm; Thermo Fisher Scientific) and separated on an Easy-Spray PepMap RSLC C18 analytical column (2 µm, 50 µm × 15 cm) maintained at 50 °C. The mobile phases consisted of buffer A (0.1% formic acid in water) and buffer B (0.1% formic acid in 80% acetonitrile), with a flow rate of 300 nL/min. The 15-min gradient was programmed as follows: 4–10% B for 2 min, 10–40% B for 11 min, 40–99% B for 0.5 min, and 99% B for 1.5 min.

Before injection, peptide samples were resuspended in 10 µL of buffer A, and 1 µL was injected for each run. Ionization was performed using nano-electrospray with a spray voltage of 1900 V and a capillary temperature of 280 °C. Data were acquired in data-independent acquisition (DIA) mode. Full MS scans covered the m/z range 380–980 with a resolution of 240,000 (at m/z 200), an automatic gain control (AGC) target of 500%, and a maximum injection time (IT) of 5 ms. MS/MS spectra were acquired in the Astral analyzer using an AGC target of 500%, an IT of 3 ms, and a normalized collision energy (NCE) of 25. The scan range was set to m/z 380–980, with 2 m/z isolation windows and optimized window placement, resulting in 299 windows per cycle.

Raw DIA data were processed using *Spectronaut* v19.1 (Biognosys AG) with the *directDIA* workflow. Spectral library generation employed the UniProt human proteome database (UP000005640_9606.fasta, March 2024; 20,418 entries), the *D. immitis* proteome database retrieved from WormBase Parasite (release WBPS19, BioProject PRJEB1797; 12,609 entries), and a dataset of common contaminants (246 entries). Trypsin/P and LysC/P were defined as proteases, allowing up to two missed cleavages. Carbamidomethylation of cysteine was set as a fixed modification, while methionine oxidation and N-terminal acetylation were defined as variable. A maximum false discovery rate (FDR) of 0.01 was applied at PSM, peptide, and protein levels. Automatic cross-run normalization was enabled, and all identified peptides contributed to protein quantification. Protein inference was performed using the *IDPicker* algorithm, and missing values were not imputed during quantification.

### Bioinformatic analysis

Differential expression analysis was performed in *Spectronaut*, considering proteins with an absolute log_2_ratio ≥ 0.58 and a *Q*-value ≤ 0.05 as significantly dysregulated. Visualization of differential expression results, including principal component analysis (PCA) and volcano plots, was conducted in R using the ggplot2 package (Wickham, 2016). Functional enrichment of differentially expressed proteins was assessed through STRING-DB v.12 (Szklarczyk et al., 2025) on the basis of Gene Ontology (GO) annotations, and enrichment results were plotted with ggplot2. Identification of angiogenesis-related proteins was performed via the Uniprot Database by consulting GO terms within the biological process category. Venn diagrams and Upset Plots were used to characterize differences in expression patterns.

## Results

### Effect of the treatments on cell viability and cytotoxity

We verified that the viability of cells exposed to VEGF-A, DiSA, DiES, DiSA + VEGF-A, and DiES + VEGF-A for 24 h and cytotoxity remained equivalent to that of untreated cultures. The number of living cells exceeded 87% in all situations, with no differences observed between treated cultures compared with nonstimulated cells. At the same time, no cytotoxic effect was observed with no significant differences between them.

### Principal component analysis and volcano plot

Proteomic analysis allowed for the identification of 420 protein groups present in all replicates of cell supernatants and 4931 protein groups present in all replicates of cell lysates. Following the exclusion of proteins related to the *D. immitis* proteome and common contaminants, 236 and 4909 protein groups from the cell supernatants and lysates, respectively, were used for subsequent analyses (Additional files 3–6: Tables S1–S4).

**Fig. 1:**
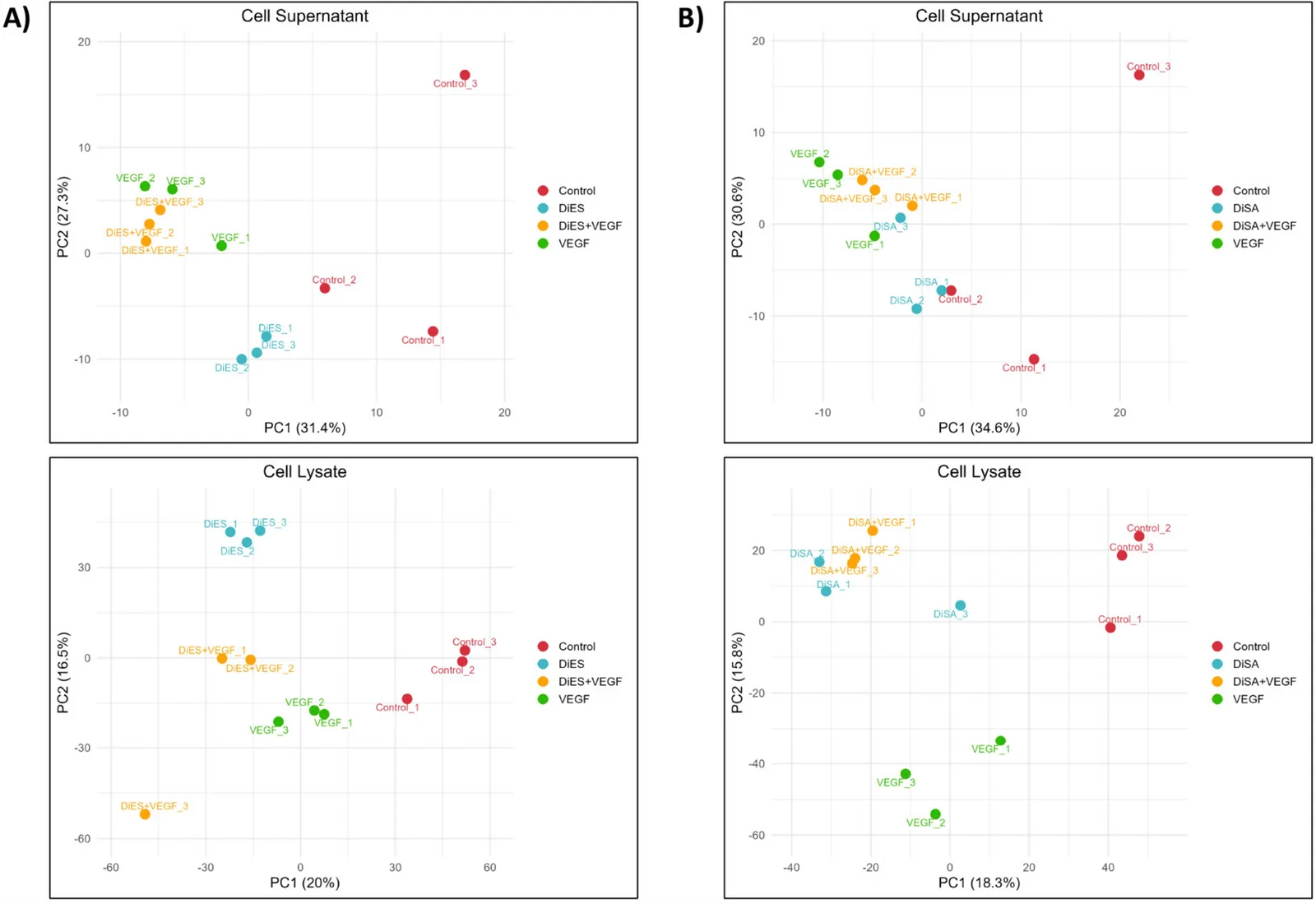
PCA analysis of proteins identified after stimulation with DiES, DiES + VEGF-A, and VEGF-A (**A**) and DiSA, DiSA + VEGF-A, and VEGF-A (**B**) in the supernatant and cell lysate. The percentage of variability explained by PC1 and PC2 are indicated on the *x* and *y* axes, respectively. PC1 and PC2 represent the first and second principal components, which capture the largest and second-largest sources of variance in the proteomic data, respectively

Subsequently, principal component analysis (PCA) was performed to evaluate differences in the proteomic profiles of cell supernatants and lysates following stimulation with DiES, DiES + VEGF-A, and VEGF-A relative to the control group, as well as differences following stimulation with DiSA, DiSA + VEGF-A, and VEGF-A relative to the control group (Fig. 1). PCA of proteins identified in the cell supernatant following stimulation with DiES, DiES + VEGF-A, VEGF-A, and the control group allowed for the identification of four distinct clusters corresponding to each treated group, with the principal component 1 (PC1) accounting for the greatest variability between groups (31.4%) and principal component 2 (PC2) accounting for 27.3%. A similar pattern was observed in the cell lysate analysis, with PC1 accounting for 20% of the variability and PC2 for 16.5%. PCA of proteins identified in the cell supernatant following stimulation with DiSA, DiSA + VEGF-A, VEGF-A, and the control group differentiated four clusters corresponding to each treatment group. PC1 contained the highest variability between groups (34.6%) and PC2 represented 30.6% of the variability; similarly, in the cell lysate analysis, PC1 represented 18.3% of the variability and PC2 15.8%. In both instances, the highest variability corresponded to proteins identified in the cell supernatant.

To identify dysregulated proteins between the different treatment groups and the control group, an absolute log_2_ratio ≥ 0.58 was used (log_2_ratio ≥ 0.58 for upregulated proteins and ≤ −0.58 for downregulated proteins), corresponding to a fold change ≥ 1.5 and a *Q*-value ≤ 0.05 (Additional files 7–8: Tables S5–S6). In the cell supernatant, treatment with DiES + VEGF-A had the greatest impact regarding the number of dysregulated proteins, altering the expression levels of 143 proteins (42 upregulated and 101 downregulated) (Additional file 1: Fig. S1). Furthermore, stimulation with DiSA + VEGF-A induced a more moderate response, altering the expression levels of 91 proteins (28 upregulated and 63 downregulated). Likewise, stimulation with antigens alone induced a lower response compared with stimulation with antigens supplemented with VEGF-A; DiES altered the expression levels of 96 proteins (58 upregulated and 38 downregulated) and DiSA altered 52 proteins (38 upregulated and 14 downregulated). Conversely, stimulation with VEGF-A resulted in the dysregulation of 108 proteins (36 upregulated and 72 downregulated).

In the cell lysate, the group treated with DiES + VEGF-A and the group treated with DiES showed a similar effect regarding the quantity of dysregulated proteins (Additional file 1: Fig. S2). However, downregulated proteins predominated in the DiES + VEGF-A treatment (163 of 272 dysregulated proteins), whereas upregulated proteins predominated in the DiES treatment (205 of 275 dysregulated proteins). On the contrary, stimulation with DiSA + VEGF-A and DiSA altered the expression levels of a smaller number of proteins (229 and 197, respectively), and upregulated proteins prevailed over downregulated ones in both treatments. Regarding the VEGF-A treatment, 317 dysregulated proteins were identified, among which downregulated proteins predominated (90 upregulated and 227 downregulated).

Additionally, changes in protein expression between stimulations with antigens supplemented with VEGF-A and stimulations with the antigen alone were analyzed. In the cell supernatant, comparing DiES + VEGF-A with DiES identified 121 dysregulated proteins: 10 more highly expressed in the DiES + VEGF-A treatment and 111 in the DiES treatment. In the cell lysate, 233 dysregulated proteins were identified: 30 more highly expressed in DiES + VEGF-A and 203 in the DiES treatment. Conversely, comparing DiSA + VEGF-A with DiSA, 71 dysregulated proteins were identified in the cell supernatant: 4 more highly expressed in DiSA + VEGF-A and 67 in the DiSA treatment; while in the cell lysate, 134 dysregulated proteins were identified: 97 in the DiSA + VEGF-A treatment and 37 in the DiSA treatment.

### Functional annotation

**Fig. 2:**
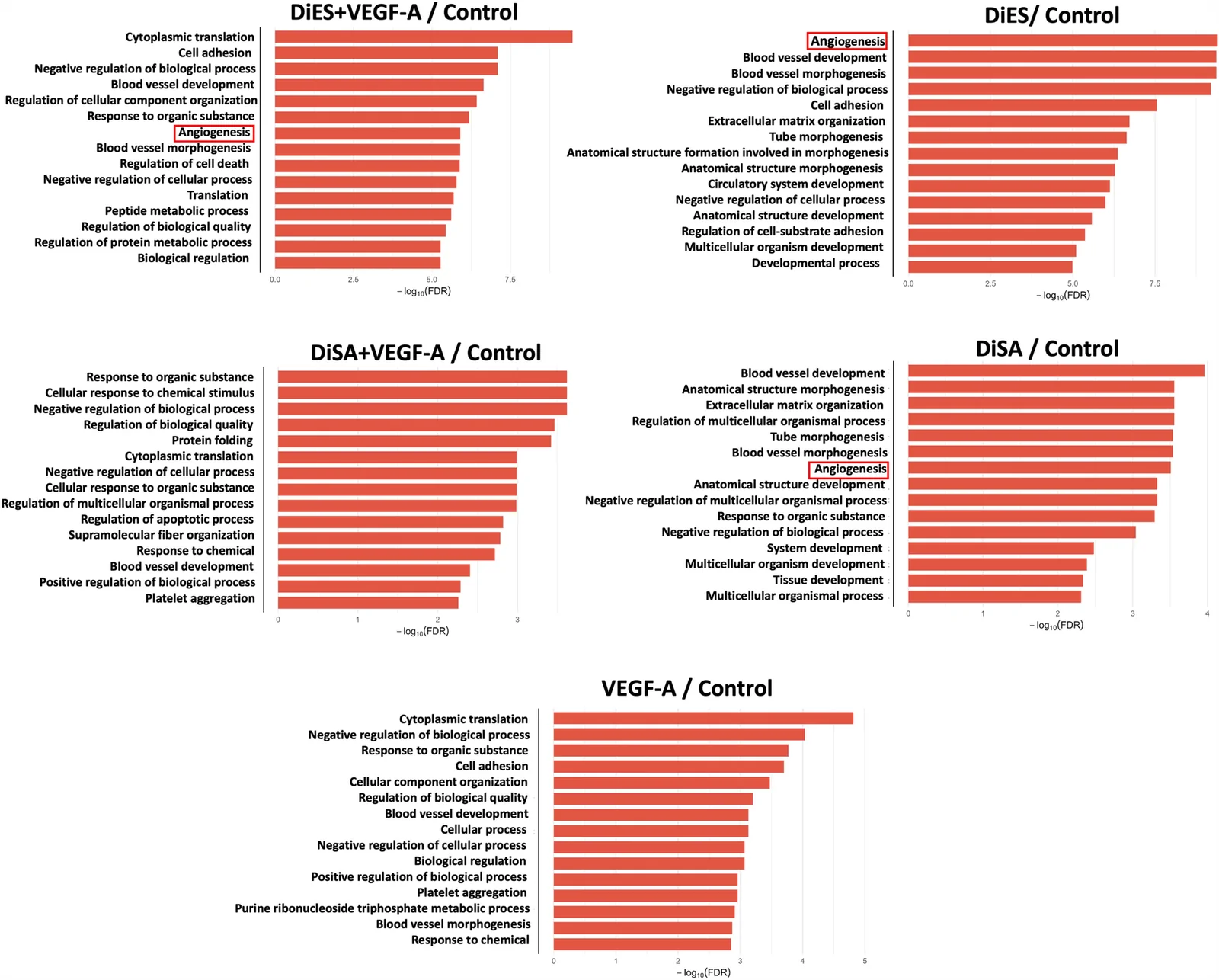
Gene Ontology enrichment analysis in the Biological Processes category of differentially expressed proteins in the cell supernatant after stimulation with different treatments compared with the control group

Gene Ontology (GO) analyses were performed on the Biological Process category for proteins differentially regulated following stimulation with DiES + VEGF-A, DiES, DiSA + VEGF-A, DiSA, and VEGF-A compared with the control group using the STRING database (Fig. 2). The GO analyses were restricted to proteins differentially regulated in the supernatant, as these more accurately reflect the response to the different treatments, unlike lysates, where the high abundance of identified proteins could mask dysregulated biological processes.

In the analysis of dysregulated proteins following stimulation with DiES + VEGF-A compared with the control group, angiogenesis was the seventh biological process with the highest level of enrichment (FDR = 1.24E−08), and following stimulation with DiES, angiogenesis was the top biological process with the highest level of enrichment (FDR = 3.94E−12). Following stimulation with DiSA, angiogenesis was less significant (FDR = 0.00031), while it was not enriched following stimulation with DiSA + VEGF-A. Following stimulation with VEGF-A, the term angiogenesis was not among the main enriched biological processes.

### Identification of proteins related to angiogenesis

To identify differentially significantly dysregulated proteins involved in the angiogenic process, the UniProt database was consulted to examine Gene Ontology annotations within the Biological Process category. A filter was applied to select proteins involved with the term “angiogenesis” for subsequent analyses.

**Fig. 3:**
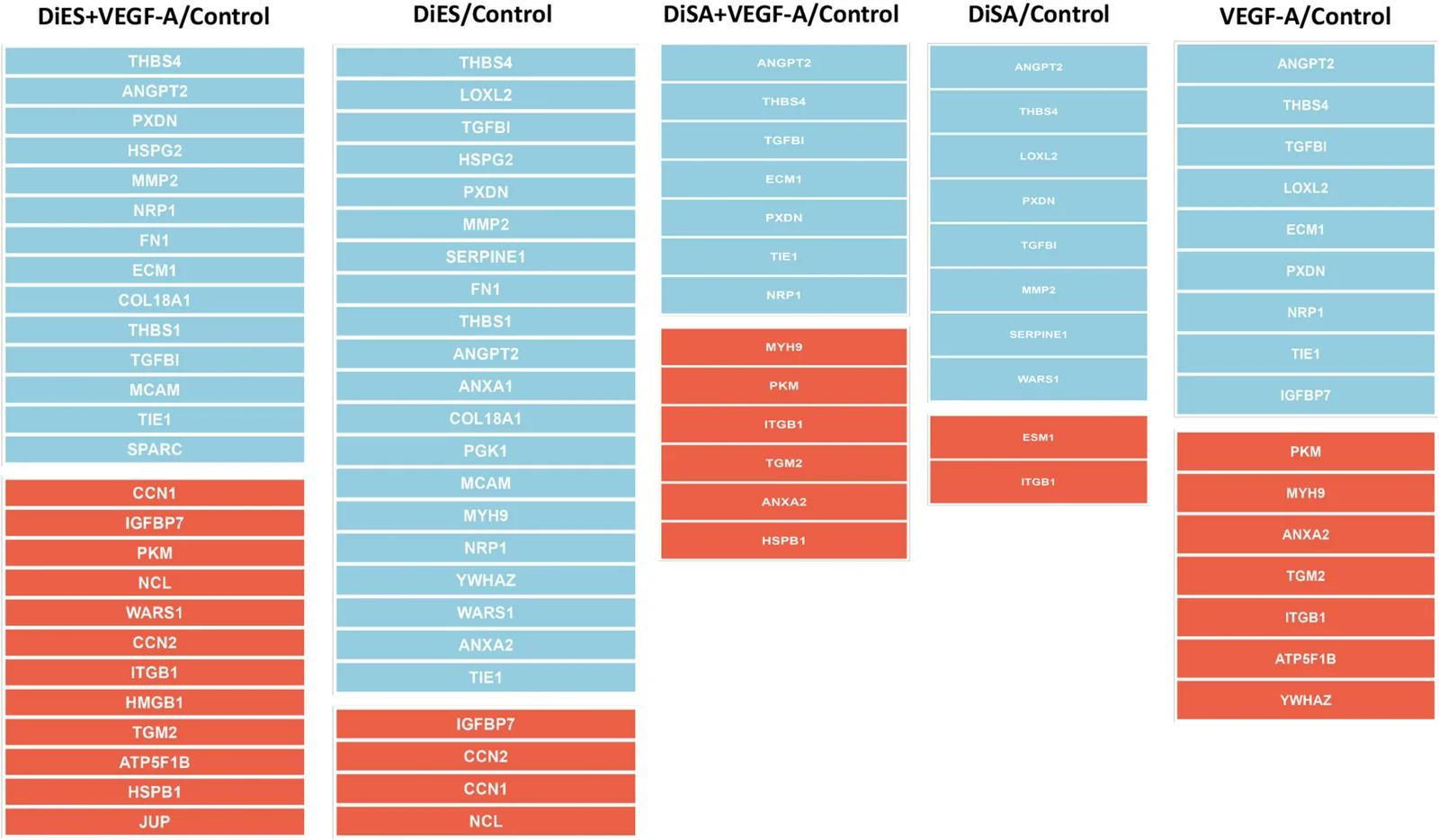
Proteins related to angiogenesis upregulated () and downregulated () after stimulation with different treatments in the cell supernatant compared with the control group

Analysis of the supernatant revealed differences in expression patterns among the different treatments compared with the control group. Stimulation with DiES + VEGF-A induced the most complex response, dysregulating a greater number of proteins related to the process, followed by stimulation with DiES. This contrasted with the DiSA treatment (with and without VEGF-A), which induced a more moderate response regarding the number of dysregulated proteins (Fig. 3).

**Fig. 4:**
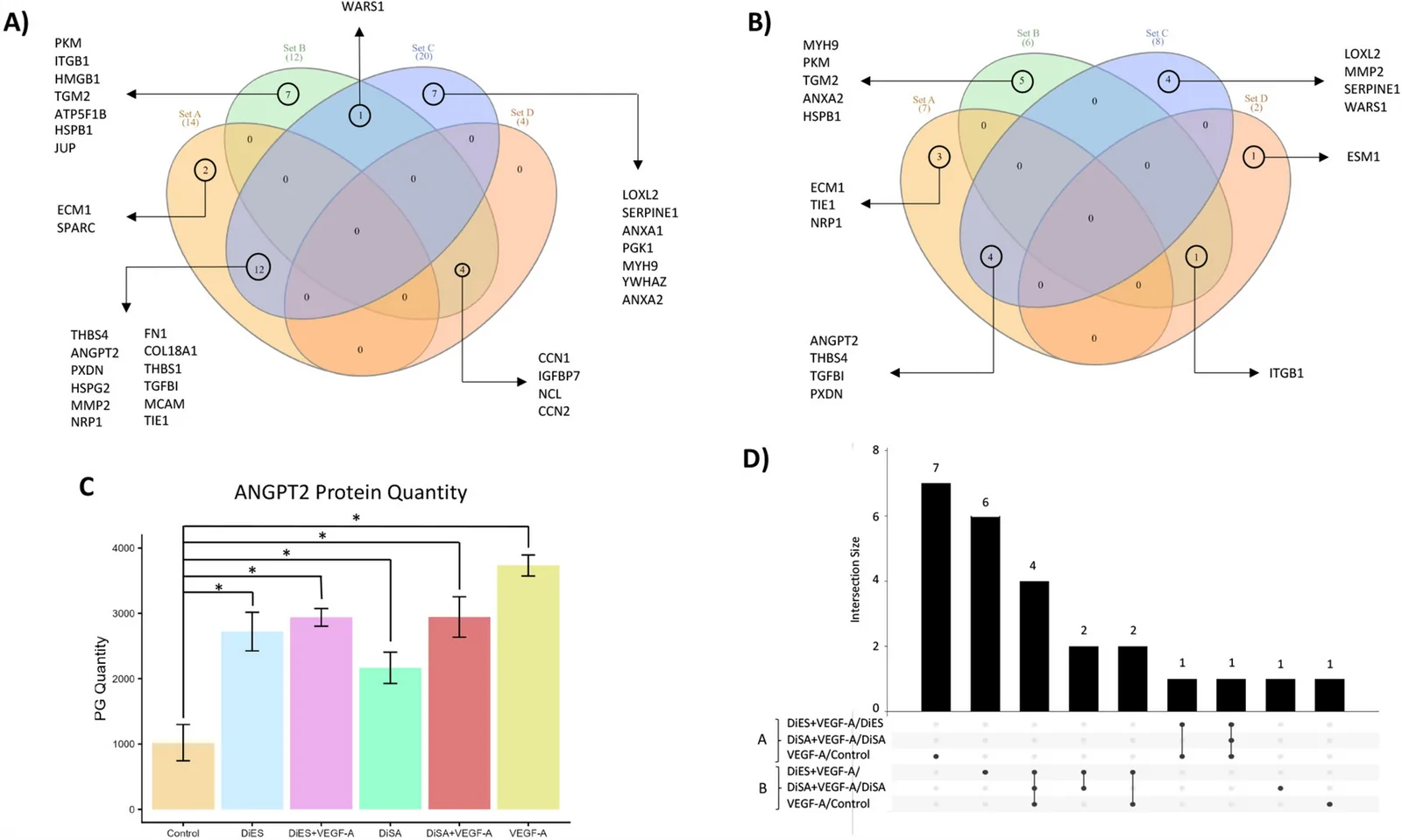
Venn diagram and UpSet plot of differentially expressed proteins after different treatments in the cell supernatant: **A** differential expression of proteins between treatment with DiES + VEGF-A and DiES (set A and C, upregulated proteins; set B and D, downregulated proteins) compared with the control group; **B** differential expression of proteins between treatment with DiSA + VEGF-A and DiSA (set A and C, upregulated proteins; set B and D, downregulated proteins) compared with the control group; **C** ANGPT2 expression levels between different treatments; **D** differences between different treatments and unstimulated (control) (**A** proteins most expressed in the numerator condition, **B** proteins most expressed in the denominator condition). *Absolute log_2_ratio ≥ 0.58 and a *Q*-value ≤ 0.05

All treatments were characterized by a significant increase in the secretion of angiopoietin-2 (ANGPT2), as well as other proteins such as thrombospondin-4 (THBS4) and peroxidasin (PXDN) (Fig. 4). Conversely, following stimulation with DiES, intracellular and membrane-localized proteins (MYH9, YWHAZ, ANXA1, ANXA2) were identified as upregulated. Furthermore, elevated levels of the anti-angiogenic proteins tryptophanyl-tRNA synthetase 1 (WARS1) and serpin family E member 1 (SERPINE1) were detected in this group. Stimulation with DiES + VEGF-A reversed this pattern, with no observed differences in the expression levels of intracellular and membrane proteins in the supernatant, and observing the downregulation of WARS1. Treatment with DiSA + VEGF-A and DiSA had a lesser effect regarding changes in the expression of proteins related to the angiogenic process; however, elevated levels of WARS1 and SERPINE1 were detected only in the DiSA treatment, whereas they remained unaltered in the DiES + VEGF-A treatment.

A comparison was performed between groups treated with antigens supplemented with VEGF-A and groups treated with the antigen alone (Fig. 4). Four common proteins were identified that were upregulated following stimulation with DiES and DiSA (ANXA2, ATP5F1B, MYH9, and TGM2), which were notably downregulated following stimulation with VEGF-A compared with the control.

**Fig. 5:**
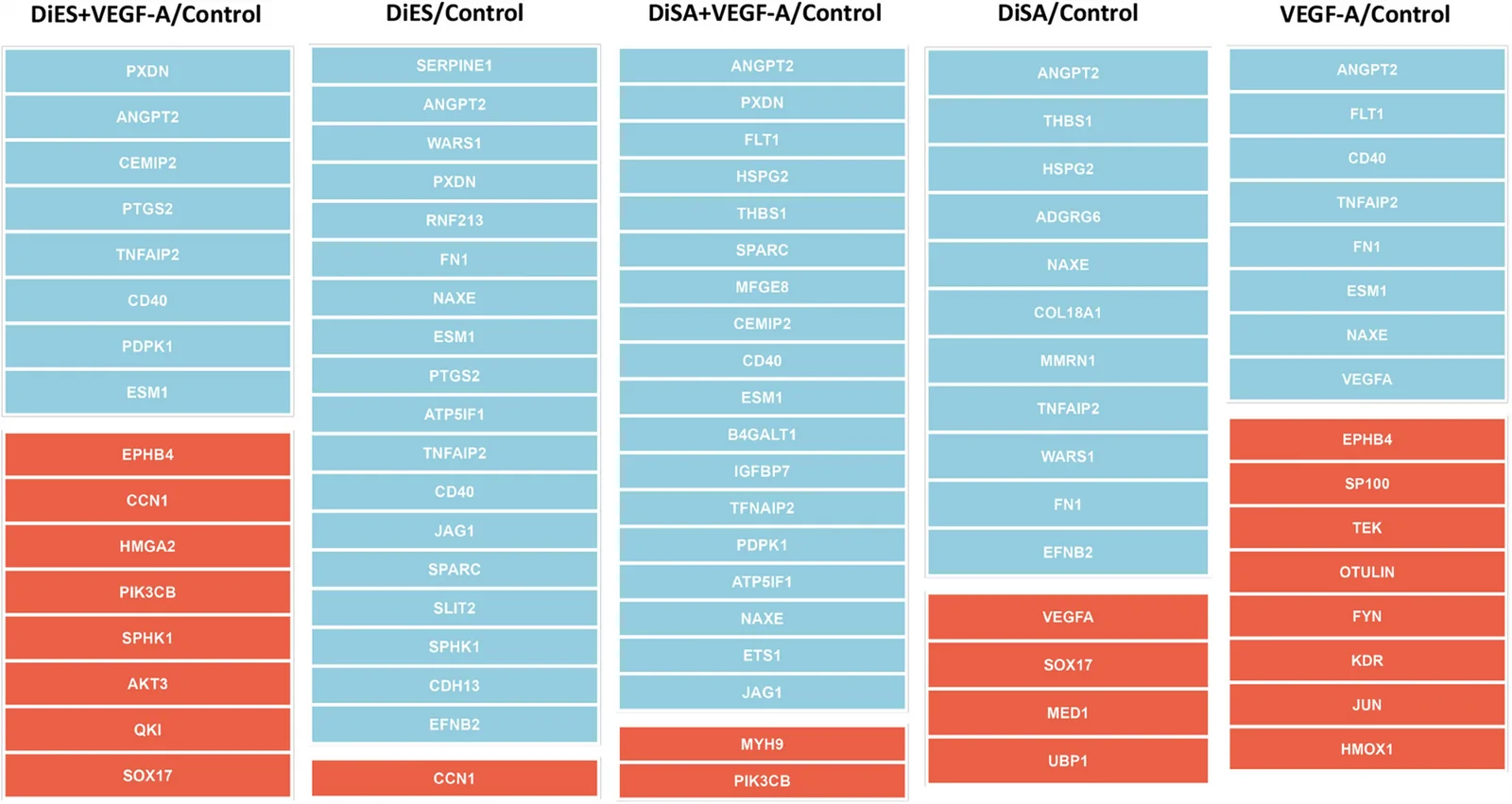
Proteins related to angiogenesis upregulated () and downregulated () after stimulation with different treatments in cell lysate compared with the control group

Further, in cell lysates, treatment with DiES produced the greatest dysregulation of angiogenic proteins (Fig. 5). Stimulation with DiES induced significant upregulated of proteins related to the cell barrier and adhesion, such as SLIT2, JAG1, or the adhesion molecule CDH13, in addition to proteins with anti-angiogenic function such as WARS1 and SERPINE1, which were also upregulated in the supernatant. In contrast, upregulated proteins of this set of vessel-stabilizing or anti-angiogenic proteins were not observed in the group treated with DiES + VEGF-A. A similar trend occurred following stimulations with DiSA + VEGF-A and DiSA, which produced an increase in the expression levels of anti-angiogenic proteins, including THBS1, IGFBP7, or WARS1.

**Fig. 6:**
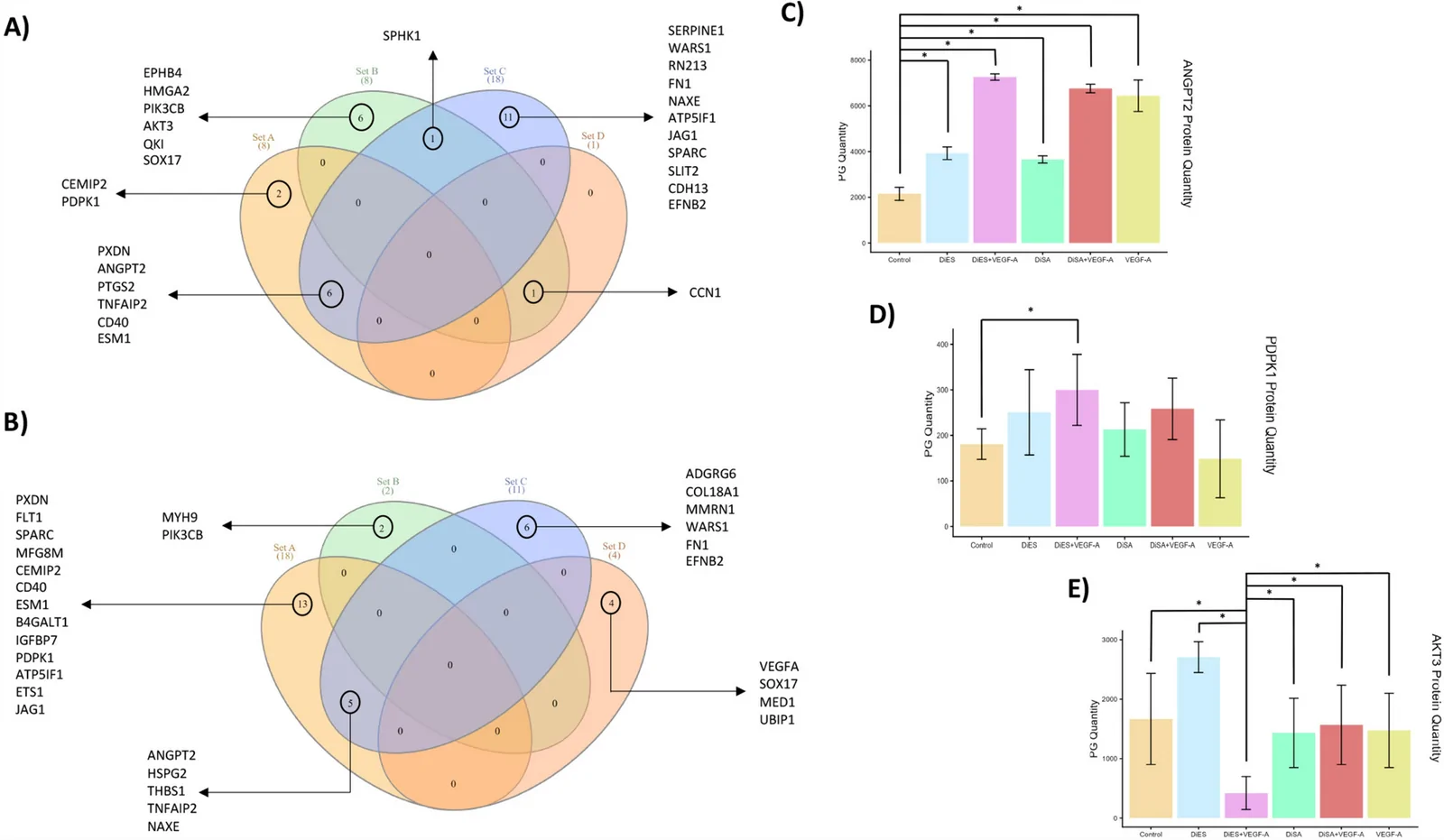
Venn diagram and Upset plot of differentially expressed proteins after different treatments in cell lysate. **A** Differential expression of proteins between DiES + VEGF-A and DiES treatment (set A and C, upregulated proteins; set B and D, downregulated proteins) compared with the control group; **B** differential expression of proteins between DiSA + VEGF-A and DiSA treatment (set A and C, upregulated proteins; set B and D, downregulated proteins) compared with the control group; **C–E** differences in the expression levels of ANGPT2, PDPK1, and AKT3 proteins, respectively. *Absolute log_2_ratio ≥ 0.58 and *Q*-value ≤ 0.05

As observed in the cell supernatants, ANGPT2 remained upregulated following stimulation with all treatments, with levels being higher following stimulation with antigens supplemented with VEGF-A than following stimulation with the antigen alone (Fig. 6). Furthermore, the protein 3-phosphoinositide-dependent protein kinase 1 (PDPK1) showed significant upregulated only following stimulation with antigens supplemented with VEGF-A, with the response being slightly higher in the DiES + VEGF-A treatment than in DiSA + VEGF-A. Finally, significant downregulation of protein kinase B (AKT) 3 was detected only in the group treated with DiES + VEGF-A compared with the rest of the experimental conditions.

## Discussion

Angiogenesis is a process consisting of the formation of new blood vessels from preexisting ones. It occurs in both normal and pathological situations in response to factors such as hypoxia, being essential in processes such as tumor growth or inflammation through the regulation of factors related to the process [19]. Studies regarding the relationship between *D. immitis* and the angiogenic process have been previously conducted. In an in vitro model, treatment with DiES + VEGF-A resulted in increased levels of pro-angiogenic factors such as VEGF-A, VEGFR2, and endoglin, as well as an increase in related cellular processes such as cell proliferation, migration, and the formation of pseudocapillaries [11]. However, treatment with DiSA induced a very mild endothelial response, increasing only VEGF-A levels and having no effect on pseudocapillary formation [12].

Our objective was to conduct a proteomic study to characterize the angiogenic response produced by endothelial cells in response to DiES and DiSA in a normoxia model, supplementing the antigens with VEGF-A to simulate the initiation of this process.

In the cell supernatant, stimulation with DiES + VEGF-A had the greatest impact regarding the quantity of dysregulated proteins related to this process. Conversely, stimulation with DiSA (with and without VEGF-A) showed a considerable reduction in the number of proteins dysregulated in association with angiogenesis compared with stimulation with DiES (with and without VEGF-A). This is reflected in the Gene Ontology analyses performed on the set of all dysregulated proteins in the supernatant following the different stimulations, in which treatment with DiES and DiES + VEGF-A enriched the angiogenic process more significantly than treatment with DiSA, whereas in the DiSA + VEGF-A treatment, it was not enriched at all.

One of the most notable findings was the upregulation of angiopoietin-2 (ANGPT2) in all treatments, in both the supernatant and cell lysates. The ANGPT/TIE axis is an endothelial cell signaling pathway that, under normal conditions, is related to the maintenance of vascular stability through the binding of the ANGPT1 ligand to its TIE2 receptor. However, in pathological situations involving endothelial activation, ANGPT2 is released; this antagonizes ANGPT1 binding to the TIE2 receptor [20–22], promoting vessel destabilization and the consequent increase in permeability necessary for the onset of angiogenesis [23]. In the presence of VEGF-A, ANGPT2 is a potent pro-angiogenic factor that acts on basal lamina remodeling as well as endothelial cell proliferation and migration; however, in the absence of VEGF-A, the protein induces cell death and vascular regression [24, 25]. This phenomenon aligns with our results, where stimulation with DiES and DiSA in the absence of VEGF-A led to increased levels of cytosolic and membrane proteins, such as annexins and MYH9, in the cell supernatant. The detection of cytosolic and membrane proteins in the medium acts as a standard marker for the loss of membrane integrity and cell lysis [26]. Furthermore, in the sole stimulation with antigens, proteins such as WARS1 and SERPINE1 were identified; these act as anti-angiogenic proteins by blocking the initiation of the process [27, 28]. Stimulation of antigens with VEGF-A reverted this pattern, with no observed increase in cytosolic and intracellular protein levels in the supernatant. Additionally, only DiES + VEGF-A stimulation reduced WARS1 levels compared with the control group.

Regarding cell lysates, DiES stimulation produced the highest quantity of dysregulated angiogenic proteins. Most were proteins related to the maintenance of vascular stability and cell quiescence, in addition to anti-angiogenic proteins such as WARS1 and SERPINE1, which were also detected in the supernatant and whose levels remained unaltered following DiES + VEGF-A stimulation. In the treatment with DiSA + VEGF-A, a greater number of upregulated proteins related to vascular stability maintenance were also detected, as well as anti-angiogenic proteins whose elevated levels in the cell lysate imply a higher production of this protein following stimulation with DiSA compared with DiES.

Furthermore, only stimulations with the antigen supplemented with VEGF-A produced a significant increase in the levels of PDPK1, a key upstream regulator of the phosphatidylinositol-3 kinase (PI3K)/AKT signaling pathway, which is known to promote endothelial cell survival, migration, and angiogenic responses in the presence of VEGF-A [29, 30]. The AKT family comprises three isoforms with distinct and sometimes opposing functions in the endothelium, including AKT1, which promotes endothelial cell growth, migration, and survival, and AKT3, which has been reported to exert inhibitory effects on these processes [31, 32]. Notably, only DiES + VEGF-A stimulation resulted in a significant reduction of AKT3 levels compared with the other experimental conditions. This shift in the balance between AKT isoforms may favor AKT1-driven signaling downstream of PDPK1, thereby enhancing pro-proliferative and pro-migratory responses in endothelial cells. In the context of *D. immitis* infection, such modulation of the PI3K/AKT pathway by parasite-derived antigens in the presence of host angiogenic factors could contribute to the vascular remodeling and endothelial proliferation described in infected animals. The ability of DiES to potentiate VEGF-A-mediated signaling while simultaneously downregulating inhibitory AKT isoforms suggests a mechanism by which parasite antigens may exacerbate angiogenic and proliferative processes within the vascular endothelium, potentially playing a role in the pathogenesis of heartworm-associated vascular lesions.

## Conclusions

Our proteomic analysis reveals that *Dirofilaria immitis* antigens differentially modulate endothelial function. While both DiES and DiSA induce vascular destabilization via ANGPT2 upregulated, their angiogenic outcomes diverge significantly. DiES, particularly with VEGF-A, acts as a potent pro-angiogenic stimulus by downregulated anti-angiogenic factors such as WARS1 and modulating the PI3K/AKT pathway through PDPK1 upregulation and AKT3 suppression, thereby prioritizing cell survival and migration. In contrast, DiSA elicits a blunted angiogenic response. Furthermore, in the absence of VEGF-A, both antigens promote vascular regression and loss of membrane integrity. These findings suggest that *D. immitis* exploits the ANGPT/TIE and PI3K/AKT signaling axes to remodel the host vasculature, providing key molecular insights into the pathophysiology of heartworm disease and potential therapeutic targets. Further research is essential to fully characterize this angiogenic response, offering new perspectives on the host–parasite interface that could improve our understanding of the disease’s inflammatory and pathogenic process.

## Supplementary Information

**Additional file 1: Figure S1**. Differential expression of proteins in the cell supernatant according to the criteria of an absolute AVG log_2_ratio ≥ 0.58 (*x*-axis) and a *Q*-value ≤ 0.05 (*y*-axis) after stimulation with the different treatments compared with the control group

**Additional file 2: Figure S2**. Differential expression of proteins in the cell lysate according to the criteria of an absolute AVG log_2_ratio ≥ 0.58 (*x*-axis) and a *Q*-value ≤ 0.05 (*y*-axis) after stimulation with the different treatments compared with the control group

**Additional file 3: Table S1**. Protein quantity of identified proteins after all treatments and control group in cell supernatants

**Additional file 4: Table S2**. Protein quantity of identified proteins after all treatments and control group in cell lysates

**Additional file 5: Table S3**. Differential expression of proteins identified in the supernatants between the different groups

**Additional file 6: Table S4**. Differential expression of proteins identified in the cell lysates between the different groups

**Additional file 7: Table S5**. Differentially up- and downregulated proteins in cell supernatants with a *Q*-value ≤ 0.05 and AVG log_2_ratio ≥ 0.58 between different groups

**Additional file 8: Table S6**. Differentially up- and downregulated proteins in cell lysates with a *Q*-value ≤ 0.05 and AVG log_2_ratio ≥ 0.58 between different groups

## Data Availability

The datasets supporting the conclusions of this article are included within the article.

## Abbreviations

PDPK1: 3-Phosphoinositide-dependent protein kinase 1
ACT: Actin
ANGPT: Angiopoietin
AGC: Automatic gain control
*Dirofilaria immitis*: *D. immitis*
DC: Detergent-compatible
DIA-LFQ: Data-independent acquisition-label-free quantification
DiES: Excretory/secretory antigens of *D. immitis*
FDR: False discovery rate
FGF: Fibroblast growth factors
FBAL: Fructose-bisphosphate aldolase
GAL: Galectin
GO: Gene Ontology
GAPDH: Glyceraldehyde-3-phosphate dehydrogenase
HUVEC: Human umbilical vein endothelial cells
IT: Injection time
LC: Liquid chromatography
MS/MS: Mass spectrometry
NCE: Normalized collision energy
PXDN: Peroxidasin
PI3K: Phosphatidylinositol-3 kinase
PCA: Principal component analysis
AKT or PKB: Protein kinase B
SERPINE1: Serpin family E member 1
DiSA: Somatic antigens of *D. immitis*
THBS4: Thrombospondin-4
TCEP: Tris(2-carboxyethyl)phosphine
WARS1: Tryptophanyl-tRNA Synthetase 1
TIE: Tyrosine kinase receptor
VEGF: Vascular endothelial growth factor
